# Same-Day Diagnosis Based on Histology for Women Suspected of Breast Cancer: High Diagnostic Accuracy and Favorable Impact on the Patient

**DOI:** 10.1371/journal.pone.0103105

**Published:** 2014-07-21

**Authors:** Maarten W. Barentsz, Hester Wessels, Paul J. van Diest, Ruud M. Pijnappel, Carmen C. van der Pol, Arjen J. Witkamp, Maurice A. A. J. van den Bosch, Helena M. Verkooijen

**Affiliations:** 1 Department of Radiology, University Medical Center Utrecht, Utrecht, The Netherlands; 2 Department of Corporate Communications, University Medical Center Utrecht, Utrecht, The Netherlands; 3 Department of Pathology, University Medical Center Utrecht, Utrecht, The Netherlands; 4 Department of Surgery, University Medical Center Utrecht, Utrecht, The Netherlands; 5 Imaging Division, University Medical Center Utrecht, Utrecht, The Netherlands; H. Lee Moffitt Cancer Center & Research Institute, United States of America

## Abstract

**Background:**

Same-day diagnosis based on histology is increasingly being offered to patients suspected of breast cancer. We evaluated to which extent same-day diagnosis affected diagnostic accuracy and patients' anxiety levels during the diagnostic phase.

**Patients and methods:**

All 759 women referred for same-day evaluation of suspicious breast lesions between November 2011–March 2013 were included. Diagnostic accuracy was assessed by linking all patients to the national pathology database to identify diagnostic discrepancies, in which case slides were reviewed. Patients' anxiety was measured in 127 patients by the State Trait and Anxiety Inventory on six moments during the diagnostic workup and changes over time (< = 1 week) were analyzed by mixed effect models.

**Results:**

Core-needle biopsy was indicated in 374/759 patients (49.3%) and in 205/759 (27%) patients, invasive or *in situ* cancer was found. Final diagnosis on the same day was provided for 606/759 (79.8%) patients. Overall, 3/759 (0.4%) discordant findings were identified. Anxiety levels decreased significantly over time from 45.2 to 30.0 (*P* = <0.001). Anxiety levels decreased from 44.4 to 25.9 (*P* = <0.001) for patients with benign disease, and remained unchanged for patients diagnosed with malignancies (48.6 to 46.7, *P* = 0.933). Time trends in anxiety were not affected by other patient or disease characteristics like age, education level or (family) history of breast cancer.

**Conclusion:**

Same-day histological diagnosis is feasible in the vast majority of patients, without impairing diagnostic accuracy. Patients' anxiety rapidly decreased in patients with a benign diagnosis and remained constant in patients with malignancy.

## Introduction

Every year, around 1.38 million women are diagnosed with breast cancer worldwide [1]. For every breast cancer case detected, many more women visit their doctor with symptoms or abnormal screening mammograms. Uncertainty about a breast cancer diagnosis is stressful. A recent paper on distress in the radiologist's waiting room showed that women waiting to undergo core needle breast biopsy experienced excessively high levels of stress and anxiety, even higher than patients waiting to undergo radiological interventions for benign or malignant conditions [2].

Stress and anxiety diminish following diagnosis, but stressful emotions persist longer among patients in whom the diagnostic trajectory had taken longer [3], [4]. Therefore, it makes sense to reduce the time of uncertainty about diagnosis in women suspected of breast cancer.

In our institution, same-day diagnosis for breast cancer was introduced in 2011 with the aim to provide ≥80% of women suspected of breast cancer with a definitive diagnosis within one day after referral. Our process combines the following features: 1) immediate appointment for the same or next working day, 2) all diagnostic interventions on the day of first visit, and 3) same day *histological* assessment of core needle biopsies.

In this study we report on a prospective evaluation of logistic feasibility, diagnostic accuracy and patient's anxiety of same-day diagnosis for breast cancer.

## Methods

This study includes all women referred to our breast clinic for evaluation of a breast lesion between November 2011 and March 2013. The study was approved by the Medical Ethics Committee of the University Medical Center Utrecht. Written informed consent was obtained from each study participant.

Same-day diagnosis was introduced in our hospital in November 2011. After referral, women with symptomatic breast lesions or abnormal screening mammograms were tentatively offered same-day diagnosis on the same or next working day. Each day several time slots were reserved for same-day diagnosis patients. Patients were first seen by a dedicated breast nurse practitioner who took the medical history and performed physical examination. Immediately after, patients went to the radiology department for mammography, followed by ultrasound examination if indicated. Immediate ultrasound guided core needle biopsy (14 gauge) was performed in patients with ultrasound detectable lesions (BIRADS III–V). In patients requiring biopsy of lesions that were visible only on mammography (e.g. microcalcifications), a biopsy could be performed within three workdays.

At the pathology department, the tissue was placed in an automated tissue processor (Peloris, Leica, Valkenswaard, The Netherlands). After approximately 100 minutes of processing time, the tissue was embedded in paraffin. In order to obtain a final diagnosis on the same-day, histologic material was sent to the pathology department fixated in formalin before 11 am. Biopsy results for histological tissue arriving after 11 am were available on the next working day.

After the visit to the radiologist, patients returned to the nurse practitioner. In case imaging and clinical examination revealed no suspicious findings, patients were informed and send home with a benign diagnosis. In case histological biopsy was performed, patients were asked to come back at the end of the day for final diagnosis.

In daily multidisciplinary breast team meetings, involving radiologists, surgeons, pathologists, radiation oncologists, oncologists, geneticists and nurse practitioners the patients' results were discussed to reach a final diagnosis and provisional treatment plan, which were communicated to the patient by the nurse practitioner. For patients diagnosed with breast cancer, an additional follow-up visit with a dedicated breast surgeon was planned within two working days.

### Data collection

For all patients with breast symptoms or abnormal screening mammograms, we prospectively collected information on age, demographics, history of breast disease, imaging (e.g. BIRADS, mammographic and ultrasound findings), clinical findings, and histology.

In terms of outcomes, we evaluated same-day diagnosis on three levels: 1) logistic feasibility, 2) diagnostic accuracy, and 3) impact on patients' stress and anxiety. Logistic feasibility was defined as the proportion of patients that we were able to offer a visit within one working day and the proportion of patients receiving a final diagnosis on the same day as the first visit. For this purpose, we prospectively recorded date of referral, proposed date of first visit, actual date of first visit, and date of final diagnosis.

In order to determine diagnostic accuracy, we linked all patients who underwent same-day diagnosis to the national pathology database (PALGA, Pathologisch-Anatomisch Landelijk Geautomatiseerd Archief) allowing a minimum follow up period of at least 3 months. PALGA captures all histological and cytological diagnoses nationwide. In case of discrepancy between the diagnosis obtained during same-day diagnosis and findings at follow up, a dedicated breast pathologist (PvD) reviewed all pathology slides. False positive findings were defined as patients with a histological diagnosis of DCIS or invasive cancer following same day diagnosis, which was not confirmed by surgery. False negative results were defined as patients diagnosed as having no abnormalities or benign disease at same day diagnosis, while at follow up the suspected lesion turned out to be malignant.

### Patients' stress and anxiety

Between October 2012 and June 2013, we measured stress and anxiety in a subgroup of patients by means of the six-item State Trait Anxiety Inventory (STAI) [5], [6]. Patients were asked to fill out this questionnaire 1) in the waiting room before intake by the nurse practitioner, 2) in the radiology waiting room prior to breast imaging, 3) in the waiting room prior to the communication of the final results, 4) one day, 5) three days, and 6) seven days after same day diagnosis. Patients also filled out the Cancer Worry Scale at intake.

### Statistics

Feasibility, false positive and false negative rates were presented as proportions with confidence intervals. STAI-scores were based on a four-point scale and the global score was calculated as the sum scores of the six-items. The sum scores were finally recalculated to a final sum score ranging from 20 to 80 to be comparable to the 40-item STAI. Changes in stress and anxiety levels over time were analyzed by a linear mixed effects model [7] with random intercept and slope for time to account for repeated anxiety scores nested within patients. Repeated anxiety scores were considered nested within patients. Fixed effects were added for moment in the diagnostic process. Stratified analyses were applied to examine whether time patterns in stress and anxiety levels varied by age, diagnosis, mean cancer worry at baseline, breast cancer history, family history of breast cancer, reason for referral and level of education.

Significant differences were defined as *P* values of 0.05 or less. All statistical analyses were performed using SPSS version 20.0 (IBM SPSS Statistics, Chicago, IL, USA).

## Results

Between November 2011 and March 2013, 759 women were referred for ‘same-day diagnosis’ to the outpatient breast clinic of the University Medical Center Utrecht. The mean age of patients was 53.7 years (range 18 to 90 years) (Table 1). A total of 291 patients (38.3%) were referred by the National Screening Program and 285 (37.5%) were referred with palpable lesions. Radiological evaluation of the lesions revealed 358 suspicious lesions (BIRADS-III or higher), and in 374 patients (49.3%) core-needle biopsy was performed (i.e. 257 under ultrasound, 101 under stereotactic, 13 under both ultrasound and stereotactic and 3 under MR-guidance). Final histopathology showed invasive or *in situ* carcinoma in 205/759 patients (27%).

**Table 1:** Baseline characteristics of all same-day diagnosis patients.

	*N* = 759	%
Female	747	98.4
Age in years (mean ± SD, range)	53.7±13.7 (18–90)	
Reason of referral		
Screening abnormality	291	38.3
Palpable lesion	285	37.5
Radiographic findings	40	5.3
Complaints	127	16.7
Second opinion	16	2.1
Positive family history of breast cancer	227	29.9
Menopausal status		
Premenopausal	287	37.8
Postmenopausal	372	49.0
Perimenopausal	79	10.4
Unknown/not applicable	21	2.8
Previous breast evaluation in hospital	217	28.6
Previous breast cancer diagnosis	53	7.0
BIRADS classification suspected lesion		
I	70	9.2
II	285	37.5
III	87	11.5
IV	161	21.2
V	110	14.5
Unknown	46	5.9
Biopsy performed		
No	367	48.4
Yes, ultrasound guided	257	33.9
Yes, stereotactically	101	13.3
Yes, both ultrasound and stereotactically	13	1.7
Yes, MR-guided	3	0.4
Other (skin/cytology)	18	2.4
*In Situ* or invasive breast cancer	205	27.0
Invasive ductal carcinoma	97	46.9
Invasive lobular carcinoma	18	8.7
Invasive ductololobular carcinoma	46	22.2
Ductal carcinoma *in situ*	27	13.0
Other (tubular, papillary)	19	9.2

Abbreviations: SD: Standard Deviation; BIRADS: Breast Imaging-Reporting and Data System; MR: Magnetic Resonance.

Information on the date of referral was available for 744/759 (98%) patients, and 655/744 (88.0%) patients were offered an appointment within one day. The proposed day of first visit and the actual day of first visit were recorded for 748/759 (98.6%) patients. Some 169/748 (22.6%) patients did not accept the opportunity to visit our clinic at the proposed date.

A final (histological) diagnosis on the same day was available for 606/759 (79.8%) patients. Reasons for longer time to diagnosis (153/579 patients) included the need for stereotactic biopsy (99/153, 64.7%), tissue not arriving at the pathology department before 11 am (*n* = 28), patients' use of anticoagulants (*n* = 7), technical problems (*n* = 5), and need for additional staining of the biopsy specimens (*n* = 4).

Review of the pathology reports within the PALGA database revealed 3/759 (0.4%) discordant findings. One false positive result was found. A patient was diagnosed with DCIS on biopsy, with only atypical ductal hyperplasia (ADH) in the resection specimen. At histological review, core needle biopsy was diagnosed as ADH. The false positive biopsy had no clinical consequences since patients with ADH undergo surgical excision.

Two false negative findings were found. One case involved a patient with clustered microcalcifications, in whom the biopsy was performed on a different cluster microcalcifications revealing ADH. During postoperative follow-up of this lesion, the other cluster was biopsied and revealed a DCIS for which the patient underwent mastectomy. This false negative result led to an unnecessary two-step intervention. The other false negative result was a patient, in whom mammographic architectural distortion with normal ultrasound findings was diagnosed as non-suspect during same day diagnosis. At six months follow-up, a stereotactic biopsy was performed showing DCIS.

For the evaluation of the impact of same-day diagnosis on stress and anxiety, 160 patients were included and response was 79.4% (127/160). The mean age of responders was 52.6 years (range 18 to 85). In 51/127 (40.2%) patients, core needle biopsy was performed and 22/127 (17.3%) patients were diagnosed with *in situ* or invasive breast cancer. Overall, anxiety scores decreased significantly over time from 45.2 to 30.0 (*P*<0.001) (Table 2). For patients with a benign diagnosis, anxiety scores decreased from 44.4 to 25.9 (*P*<0.001) and for patients with a diagnosis of breast cancer, anxiety did not decrease (48.6 to 46.7, *P* = 0.933). Stratification showed that age (<65 or ≥65 years), baseline cancer worry score (<7 or ≥7), breast cancer history, family history of breast cancer, reason for referral (palpable lesions versus lesions detected by the National Screening Program), and level of education were not associated with differences in anxiety decrease (Table 2).

**Table 2:** Impact of same-day diagnosis on stress and anxiety, measured by the six-item State Trait Anxiety Inventory (STAI).

Mean STAI scores (SE)	STAI 1 before intake	STAI 2 before imaging	STAI 3 before diagnosis	STAI 4 1 day after dx	STAI 5 3 days after dx	STAI 6 7 days after dx	Overall *P-*value*
All patients (n = 127)	45.2 (1.2)	42.9 (1.2)	38.4 (1.2)	32.2 (1.3)	30.7 (1.3)	30.0 (1.5)	<0.001
Benign disease (n = 105)	44.4 (1.3)	42.1 (1.3)	36.5 (1.2)	28.2 (1.3)	26.8 (1.3)	25.9 (1.3)	<0.001
Malignant disease (n = 22)	48.6 (2.7)	46.4 (2.7)	47.7 (2.7)	48.6 (2.7)	46.6 (2.5)	46.7 (2.2)	0.933
No biopsy performed (n = 76)	45.7 (1.4)	42.8 (1.4)	34.3 (1.4)	26.3 (1.5)	25.3 (1.4)	25.5 (1.5)	<0.001
Core needle biopsy performed (n = 51)	44.4 (1.9)	43.0 (1.9)	44.8 (1.9)	40.8 (2.1)	38.5 (2.1)	36.1 (2.3)	0.005
Score CWS <7 (n = 75)^a^	43.3 (1.6)	41.6 (1.6)	38.8 (1.6)	32.5 (1.7)	31.2 (1.7)	30.2 (1.7)	<0.001
Score CWS ≥7 (n = 43)	48.3 (1.8)	45.2 (1.8)	37.7 (1.8)	31.5 (2.0)	29.4 (2.2)	29.5 (2.9)	<0.001
Age <65 years (n = 104)	45.1 (1.3)	42.6 (1.3)	37.8 (1.3)	30.8 (1.4)	28.4 (1.4)	28.4 (1.6)	<0.001
Age ≥65 years (n = 23)	45.3 (2.6)	44.1 (2.6)	41.4 (2.7)	37.2 (2.7)	38.5 (3.1)	33.9 (3.6)	0.001
No history of breast cancer (n = 120)	45.7 (1.3)	43.0 (1.3)	38.6 (1.3)	32.4 (1.3)	31.0 (1.4)	30.1 (1.6)	<0.001
History of breast cancer (n = 7)	36.2 (3.5)	41.4 (3.5)	35.2 (3.5)	28.5 (3.8)	25.8 (3.6)	28.0 (3.6)	0.024
Negative family history (n = 82)	46.5 (1.4)	44.5 (1.4)	39.6 (1.4)	33.1 (1.6)	30.7 (1.7)	30.8 (2.0)	<0.001
Positive family history (n = 45)	42.7 (2.2)	40.0 (2.1)	36.3 (2.1)	30.5 (2.3)	30.5 (2.2)	28.4 (2.3)	<0.001
Palpable lesion/complaints (n = 79)	44.1 (1.4)	42.0 (1.4)	36.2 (1.4)	28.7 (1.5)	26.9(1.5)	27.2 (1.9)	<0.001
Lesions detected by the Screening Program (n = 48)	47.1 (2.1)	44.4 (2.1)	42.1 (2.1)	37.8 (2.2)	37.9 (2.3)	34.6 (2.2)	<0.001
Education^b^: Low (n = 19)	48.2 (3.1)	46.3 (3.0)	44.6 (3.0)	34.8 (3.2)	33.5 (3.4)	33.4 (4.0)	<0.001
Medium (n = 25)	43.0 (2.4)	39.1 (2.4)	34.9 (2.4)	30.9 (2.3)	28.8 (2.3)	26.8 (2.3)	<0.001
High (n = 41)	46.5 (2.2)	44.1 (2.2)	40.7 (2.2)	31.8 (2.2)	31.8 (2.1)	30.8 (2.2)	<0.001

Abbreviations: STAI, State Trait and Anxiety Scores; SE, Standard Error; dx, diagnosis; CWS, Cancer Worry Scale.

*P-values from mixed model analysis indicating the significance of decrease in anxiety over time.

a Nine patients did not fill in the cancer worry scale.

b Level of education was unknown in 42 patients.

## Discussion

This study shows that same-day diagnosis, including histologic biopsy, is feasible in the vast majority of patients. We were able to offer 655/744 (88%) patients an appointment for evaluation of their breast lesion within one day after referral, with all investigations planned on the same day. We provided a final, often histologically proven, diagnosis within one day in almost 80% of patients. Same-day diagnosis with histological tissue assessment did not have a negative impact on diagnostic accuracy, as in only 3/759 (0.4%) patients an incorrect diagnosis was made. Evaluation of patients' stress and anxiety showed that anxiety rapidly decreased in patients with a benign diagnosis. Anxiety of patients with malignancies did not change over time.

A unique aspect of our study is that we combine rapid referral (with 88.0% patients offered an appointment within one day) with rapid diagnostic work up and rapid histological tissue assessment. Same-day histological assessment is relatively new. Bulte *et al.* [8] were able to provide a conclusive diagnosis within one day in 65% of patients undergoing ultrasound-guided biopsy. Sensitivity in their study was 96.9% for invasive and *in situ* carcinomas and specificity was 99.4%. We were able to provide a conclusive diagnosis within one day in 229/374 (61.2%) patients undergoing core-needle biopsy (either ultrasound-guided, stereotactic or MR-guided). Sensitivity of the histological biopsies was 99.5% and specificity 99.4%. These results are similar to studies on core-needle biopsies in a non-same-day setting, reporting sensitivities of 93.1% and 96.3% and specificities of 88.3% and 100% [9], [10].

We are the first to monitor patients' anxiety repeatedly during the diagnostic process of same-day diagnosis by validated questionnaires [5], [6]. Others have analyzed the impact of same-day diagnosis on patients' anxiety [3], [11], [12]. In these studies, a final diagnosis of the suspicious breast lesions was obtained with fine needle aspiration. Dey *et al.* [3] compared a one-stop breast clinic with a dedicated breast clinic and found that patients attending the one-stop clinic were less anxious 25 hours after visit, but not after 3 weeks or 3 months. Harcourt *et al.* [11] compared a one-stop system with a conventional system. Levels of anxiety were measured before diagnosis, 6 days after diagnosis and 8 weeks after diagnosis. At the first visit, levels of anxiety were high in both groups. Six days later, the one-stop group showed significant reduction in anxiety levels; however, women with cancer were more distressed than those still awaiting diagnosis. Eight weeks after diagnosis, a rapid cancer diagnosis was associated with higher levels of depressive symptoms [11]. Ubhi *et al.* [12] measured anxiety in patients undergoing fine needle aspiration of a symptomatic breast lesion. Effects of immediate communication of results were compared with delayed communication. Anxiety at baseline was high in both groups. A malignant diagnosis resulted in higher post-communication anxiety compared with benign results. Immediate communication of benign results was associated with a significantly greater fall in STAI-scores. For assessment of breast lesions, several authors advocate the use of histological biopsies [13]–[16]. In a systematic review, Willems *et al.* [14] showed that CNB had a higher success rate than FNAC (99% versus 60–74%, respectively) and higher specificity (86–100% with CNB versus 48–100% with FNAC). Another advantage of CNB over FNAC is the ability to provide immunohistochemical and molecular profiling of tumor samples, which has become more important due to the increase in neo-adjuvant treatment.

No subgroup could be identified based on patient characteristics that would not benefit from same-day diagnosis of suspicious breast lesions in terms of reducing anxiety. However, same-day diagnosis might not be suitable for all cancer types. Some patient categories might not experience high levels of stress and anxiety, while for other categories rapid cancer diagnosis could have a detrimental effect on patients' anxiety. Diagnosis related stress levels vary largely by tumor type. Patients suspected of prostate or testicular cancer experience lower levels of stress, while patients suspected of lung or breast cancer experience very high stress levels [17], [18]. Future studies are needed to evaluate which tumor types are best suited for same-day diagnosis.

Patients with a suspicion of breast cancer are suited for same-day diagnosis and especially patients with a benign diagnosis benefit from the rapid assessment. Since about 1 in 10 women with an abnormal mammogram or palpable lump actually has invasive breast cancer [19], many patients may benefit from same-day diagnosis in terms of reduction in anxiety.

In conclusion, same-day diagnosis including histologic biopsy is feasible in the vast majority of patients. A conclusive diagnosis could be provided the same day of first visit in almost 80% of patients. Rapid assessment did not have negative impact on diagnostic accuracy. Patients' anxiety rapidly decreased with a benign diagnosis and did not change in patients with a malignancy.
